# The Safety Profile of Vitamin D Supplements Using Real-World Data from 445,493 Participants of the UK Biobank: Slightly Higher Hypercalcemia Prevalence but Neither Increased Risks of Kidney Stones nor Atherosclerosis

**DOI:** 10.3390/nu16142251

**Published:** 2024-07-12

**Authors:** Sha Sha, Miriam Degen, Tomislav Vlaski, Ziwen Fan, Hermann Brenner, Ben Schöttker

**Affiliations:** 1 Division of Clinical Epidemiology and Aging Research, German Cancer Research Center (DKFZ), 69120 Heidelberg, Germany; sha.sha@dkfz-heidelberg.de (S.S.); miriam.degen@dkfz-heidelberg.de (M.D.); tomislav.vlaski@dkfz-heidelberg.de (T.V.); ziwen.fan@dkfz-heidelberg.de (Z.F.); b.schoettker@dkfz-heidelberg.de (B.S.); 2 Faculty of Medicine, University of Heidelberg, 69115 Heidelberg, Germany; 3 Division of Preventive Oncology, German Cancer Research Center (DKFZ) and National Center for Tumor Diseases (NCT), 69120 Heidelberg, Germany; 4 German Cancer Consortium (DKTK), German Cancer Research Center (DKFZ), 69120 Heidelberg, Germany

**Keywords:** vitamin D supplementation, serum 25-hydroxyvitamin D levels, adverse events, hypercalcemia, kidney stone risk, atherosclerosis, real-world evidence

## Abstract

**Background**: Potential calcium-related adverse events of vitamin D supplement use have not been addressed in large-scale, real-world data so far. **Methods:** Leveraging data from the UK Biobank, encompassing 445,493 individuals aged 40–69, we examined associations of high 25-hydroxyvitamin (25(OH)D) levels ≥ 100 nmol/L and vitamin D supplementation with hypercalcemia (serum calcium > 2.6 mmol/L), kidney stones, and atherosclerosis assessments (pulse wave arterial stiffness index and carotid intima-medial thickness). Regression models were comprehensively adjusted for 49 covariates. **Results:** Approximately 1.5% of the participants had high 25(OH)D levels, 4.3% regularly used vitamin D supplements, and 20.4% reported regular multivitamin use. At baseline, the hypercalcemia prevalence was 1.6%, and 1.1% was diagnosed with kidney stones during follow-up. High 25(OH)D levels were neither associated with calcium-related adverse events nor atherosclerosis assessments. Vitamin D and multivitamin supplementation were associated with an increased prevalence of hypercalcemia (odds ratios and 95% confidence intervals: 1.46 [1.32–1.62] and 1.11 [1.04–1.18], respectively) but were neither associated with atherosclerosis nor future kidney stones. **Conclusions:** High 25(OH)D levels observable in routine care were not associated with any adverse outcome. Vitamin D users have a slightly higher prevalence of hypercalcemia, possibly due to co-supplementation with calcium, but without a higher atherosclerosis prevalence or risk of kidney stones.

## 1. Background

Vitamin D, a critical regulator of calcium homeostasis and bone health, has garnered substantial attention in recent years for its multifaceted role in human physiology. The active hormone derived from vitamin D, known as 1,25-dihydroxyvitamin D (1,25(OH)_2_D_3_), has the capacity to bind to the vitamin D receptor found on a wide array of cells throughout the human body, including bones, muscles, immune cells, and the kidney [1,2,3,4,5,6]. Beyond its established influence on bone health, vitamin D has been found to be associated with a range of health benefits [1,3,4,5,7,8,9]. Media reports on the health effects of vitamin D have led to a strong public interest in the intake of vitamin D supplements, often without prescription or even knowledge by general practitioners. There are concerns regarding the safety of this widespread vitamin D supplement use in the public due to the profound influence of vitamin D on calcium absorption and utilisation, which might lead to hypercalcemia. This, in turn, could precipitate the formation of kidney stones, a process intricately associated with calcium regulation [10,11]. Hence, there is keen interest in exploring whether routine vitamin D supplementation in the general population is associated with adverse events related to hypercalcemia, such as kidney stones, in large real-world data.

Furthermore, in consideration of the potential deleterious impact of elevated blood calcium levels on arterial stiffness and atherosclerosis [12,13], our investigation extended its scope to evaluate the cross-sectional association of high 25(OH)D status and vitamin D supplementation with two surrogate markers of atherosclerosis-pulse wave arterial stiffness index (PASI) and carotid intima-medial thickness (CIMT).

In summary, this study will utilise real-world data from the large, population-based United Kingdom (UK) Biobank to investigate whether high serum 25(OH)D levels and vitamin D supplement use are cross-sectionally associated with hypercalcemia, PASI, and CIMT and to investigate the longitudinal associations with kidney stones.

## 2. Materials and Methods

### 2.1. Data Source

This study used data from the UK Biobank, a comprehensive prospective cohort comprising over 500,000 participants from the UK aged between 40 and 69 years at enrolment [14]. From 2006 to 2010, biomedical information was systematically collected through the 22 assessment centres situated across England, Scotland, and Wales [14]. During the assessment visit, participants provided electronically signed consent, a filled touch-screen questionnaire, a brief interview, and a broad spectrum of physical and functional assessments [14,15]. Subsequent follow-up data on health outcomes were obtained through linkages to multiple healthcare records, encompassing the UK National Health Service (NHS) data, primary care records, primary care data, cancer screening data, and disease-specific registers [16].

### 2.2. Vitamin D Status

The quantification of 25(OH)D concentrations was conducted utilising the Chemiluminescent Immunoassay on the DiaSorin Liaison XL platform (manufactured by Diasorin S.p.A) and additionally verified through the RIQAS Immunoassay Specialty I scheme [17]. Vitamin D deficiency and insufficiency were classified according to the thresholds recommended by the Institute of Medicine in the US, wherein serum 25(OH)D concentrations below 30 nmol/L and between 30 and <50 nmol/L are labelled as vitamin D deficiency and insufficiency, respectively [18]. Adequate vitamin D status was delineated by 25(OH)D levels ranging from 50 to <100 nmol/L. To assess the potential for vitamin D overdose, we considered 25(OH)D concentrations of ≥100 nmol/L as indicative of high vitamin D status. The threshold was chosen based on the suggested “optimal range” of 75–100 nmol/L for multiple health endpoints [19,20].

### 2.3. Vitamin D Supplement Use

The data regarding the usage of vitamin D and multivitamin supplements were extracted from the baseline questionnaire administered by the UK Biobank, utilising the query (Data Field 6155): “Do you regularly take any of the following?”. The available response options of “Vitamin D” and “Multivitamins with or without minerals” were extracted for analyses. Of the individuals who indicated regular consumption of vitamin D supplements, 16.7% obtained them through prescriptions, and 83.3% acquired them over-the-counter (OTC).

The exact vitamin D doses of the products used by the UK Biobank’s study participants are unknown, but it can be stated that most multivitamin products contain the recommended daily allowance, which is 400 IU of vitamin D per day. Vitamin D-specific OTC products usually contain doses of 800 IU—2000 IU vitamin D per capsule/tablet. Prescribed vitamin D products are usually used with daily doses between 1000 IU and 4000 IU vitamin D per day or equivalent weekly single doses (e.g., 20,000 IU once per week).

### 2.4. Study Outcomes

The study examined four outcomes: hypercalcemia, PASI, and CIMT in cross-sectional analyses as well as kidney or ureteral calculus in longitudinal analysis. The latter outcome is abbreviated as “kidney stones” in the following.

#### 2.4.1. Hypercalcemia

Hypercalcemia was defined as a serum calcium concentration (Data Field 30680) surpassing 2.60 mmol/L [21,22]. The assessment of serum calcium levels employed Arsenazo III analysis conducted on a Beckman Coulter AU5800 during the initial assessment visit [23].

#### 2.4.2. PASI

Pulse wave velocity (PWV) was measured with the PulseWave PCA2 device (CareFusion, San Diego, CA, USA) during the initial visit to the UK Biobank assessment centre using the pulse waveform obtained from the finger utilising an infrared sensor (Data Field 21021) [24,25]. The configuration of the volume waveform in the finger is intricately associated with the temporal dynamics of pulse wave propagation through the arterial network in the lower extremities and its subsequent rebound to the finger [24,25]. The apparatus was affixed to a digit, with measurements conducted over a period of 10–15 s. The stiffness index in metres per second (m/s) was derived from the interval between consecutive peaks of the waveform and normalised based on the height of individuals [24].

Given the absence of a consensus on PASI cut-offs to delineate normalcy and abnormality, we referenced the normal range from the device manual, which provided a normal range of stiffness index based on subject ages [26]. PASI values exceeding the upper limit of the age-specific normal stiffness index range were classified as an adversely high PASI. The equation for calculating the upper limit of the normal range was [26]:

Cut-off (adversely high PASI) = 0.1663 × age + 4.3858.

#### 2.4.3. CIMT

CIMT serves as a non-invasive method to assess subclinical changes indicative of cardiovascular disease. In the UK Biobank, CIMT measurements were documented during imaging visits commencing in 2014 (Data Fields 22670-22681) [27]. Ultrasound measurements were conducted on the far wall of the distal common carotid artery, with recordings taken at two angles on each side (left and right). Automated software facilitated the capture of images and measurements of the minimum, mean, and maximum intima-media thickness.

In this study, the average of four mean measures (two from each of the left and right carotid arteries) was computed to determine the mean CIMT value. An adversely high CIMT was defined as an average mean CIMT exceeding 0.9 mm, as recommended by the European Society of Hypertension/European Society of Cardiology hypertension guideline [28].

#### 2.4.4. Kidney Stones

Follow-up data pertaining to the initial diagnosis of kidney stones till 31 October 2022 was gathered through a variety of sources, including the death register, primary care records, hospital admission data, and self-reported information as well as amalgamated data from multiple sources within the UK Biobank using the 10th revision of the International Statistical Classification of Diseases (ICD-10) code N20 “calculus of kidney and ureter” (Data Field 132037) [29,30]. Data obtained solely from self-reports were censored during the follow-up in the analyses.

### 2.5. Covariates

The study incorporated factors previously identified to be statistically and independently associated with either vitamin D deficiency (n = 48) or vitamin D supplementation use (n = 49) from earlier analyses of the UK Biobank cohort [31]. These encompass sociodemographic factors and lifestyle factors (e.g., smoking, alcohol drinking, physical activities, and dietary preferences for food items with high vitamin D content) and a broad spectrum of biomarkers and diseases (e.g., cancer, coronary heart diseases [CHD] and diabetes) as well as vitamin D-specific factors, such as season of blood draw and geographic latitudes. Notably, we also included hyperparathyroidism diagnosed prior to or at the study baseline (Data Field 130723) as an additional covariate in all analyses. Both hyperparathyroidism and cancer are significant aetiologies of hypercalcemia [32,33]. The baseline distributions of all covariates in the population analysed are presented in Supplemental Table S1. Detailed descriptions of the assessment methods of the covariates have been outlined in a previous publication [31].

### 2.6. In- and Exclusion Criteria

**Figure 1:**
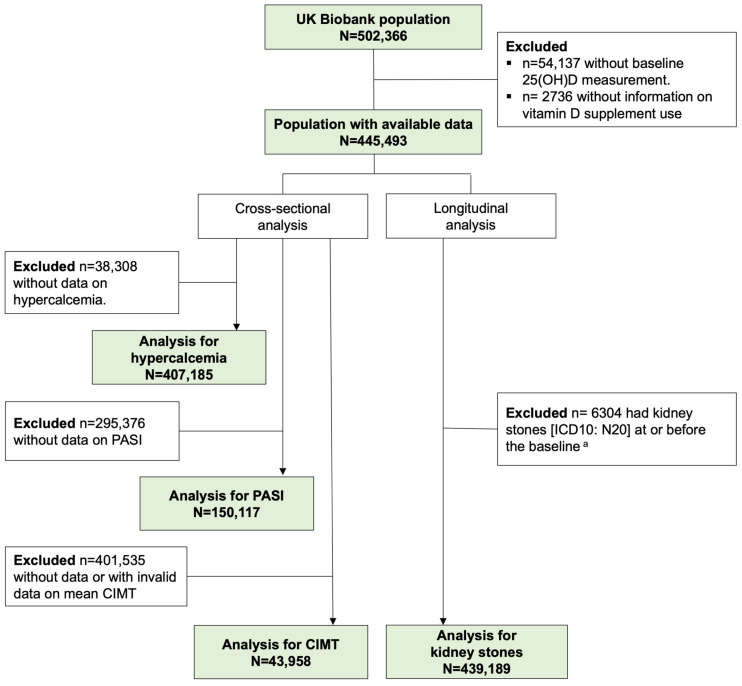
A flow chart of the study population inclusion and exclusion. Abbreviation: 25(OH)D: 25-hydroxyvitamin D, CIMT: carotid intima medial thickness, ICD-10: the 10th revision of the international classification of disease, PASI: pulse wave arterial stiffness index. ^a^ Sourced from self-reported, primary care, hospital admission, and death register data.

From the total population of n = 502,366 in the UK Biobank database, we excluded individuals without serum 25(OH)D measurements at baseline as well as those without information on vitamin D or multivitamin supplement use, resulting in a population of n = 445,493 available for the subsequent analyses (see flow chart, Figure 1). Due to variations in data availability for each outcome, individuals with missing data for the corresponding outcomes were further excluded from the cross-sectional analyses in the corresponding analytical dataset of hypercalcemia, PASI, and CIMT. Therefore, n = 407,185, n = 150,117, and n = 43,958 participants were included in the three analytical datasets, respectively. In the longitudinal analysis, individuals with a history of kidney stones at or before baseline were excluded, and n = 439,189 individuals were included in the analysis for this outcome.

### 2.7. Statistical Analyses

#### 2.7.1. General Remarks

Statistical analyses were conducted using SAS statistical software (version 9.4, SAS Institute, Inc., Cary, NC, USA). The assumption of proportional hazards was assessed using Schoenfeld residuals, revealing no violations of this assumption. All statistical tests were two-tailed, with a significance level set at α = 0.05. Multiple imputation with the Markov chain Monte Carlo method was employed to address missing data, generating five imputed datasets [34,35]. Analytical outcomes from imputed datasets were amalgamated using the SAS procedure ‘PROC MIANALYZE’.

#### 2.7.2. The Association of 25(OH)D Levels with the Study Outcomes

We used logistic regression to conduct the cross-sectional analyses examining the association of vitamin D deficiency and vitamin D insufficiency and high vitamin D status with reference to adequate vitamin D status with hypercalcemia, adversely high PASI and CIMT. Cox proportional hazard regression was used to assess the longitudinal association with kidney stones. Two distinct models were applied: model 1, which was adjusted for age and sex, and model 2, which was adjusted for all covariates (n = 49) previously identified as determinants of vitamin D deficiency (please see legend of Table 2) [31].

Furthermore, dose–response curves of 25(OH)D levels with the four outcomes were generated using restricted cubic splines (RCS) with three knots positioned at the 25th, 50th, and 75th percentiles of 25(OH)D values and full adjustment for 49 covariates [36,37]. A reference point of 75 nmol/L for 25(OH)D was used.

#### 2.7.3. The Association of Vitamin D Supplement Use with the Study Outcomes

In tandem with 25(OH)D status, logistic regression and Cox proportional hazards regressions were utilised to investigate the association of vitamin D supplements and multivitamin use with the study outcomes in cross-sectional and longitudinal analyses, respectively. The covariates present in the full models were substituted with the array of the factors previously identified as determinants of vitamin D supplement use (n = 50, see legend of Table 2).

#### 2.7.4. Subgroup Analyses

Subgroup analyses were conducted to explore the association of 25(OH)D levels and vitamin D supplement use with hypercalcemia and kidney stones across different demographic strata, including age groups (<60/≥60 years old), sexes (female/male), and levels of kidney function categorised by the estimated glomerular filtration rate (eGFR): <60, 60–<90, and ≥90 mL/min/1.73 m^2^.

## 3. Results

### 3.1. Description of the Study Population

Table 1 provides the overview of the characteristics of the available population (n = 445,493) in the UK Biobank. The median age was 58 (interquartile range [IQR]: 50; 63) years at the time of enrolment. Slightly more females (53.6%) than males participated in the study. Two-thirds (66.7%) of the population were either overweight or obese, and almost half of the participants (45.1%) had ever smoked during their life-time. Approximately one-third (31%) of the population were alcohol abstainers, while 12% had a high level of alcohol consumption per day. At the study baseline, over a quarter (26.9%) of the population had hypertension, while diabetes (5.0%), CHD (4.7%), hyperparathyroidism (0.1%), and an impaired eGFR < 60 mL/min/1.73 m^2^ (2.3%) were less frequent. The median number of chronic diseases per person was 2 (IQR: 1; 3). The distribution of all covariates considered in this study is provided in Supplemental Table S1.

In terms of the study outcomes, the median serum calcium concentration was 2.37 mmol/L (IQR: 2.31; 2.43 mmol/L), with hypercalcemia (>2.6 mmol/L) identified in 1.6% of the total population. The prevalence of hypercalcemia was twofold higher in females compared to males (2.0% versus [vs.] 1.0%). Over the median follow-up of 12.8 years, approximately 1.1% of the population had a diagnosis of kidney stones. Males had a twice-as-high incidence of kidney stone formation compared to females (1.6% vs. 0.8%). The median values for PASI and CIMT were 9.0 (IQR: 6.9; 11.1) and 0.67 (IQR: 0.60; 0.76), respectively, and adversely high PASI and CIMT were observed for 7.6% and 6.2% of the population with the data available, respectively.

A considerable proportion of the study population exhibited either vitamin D deficiency (21.0%) or insufficiency (34.3%), while only approximately 1.5% of the population had high 25(OH)D levels of 100 nmol/L and above. Very high 25(OH)D levels > 125 nmol/L were even rarer, with a prevalence of 0.2% (n = 803 participants). Almost no one (n = 6) had 25(OH)D concentrations >250 nmol/L. Notably, only 4.3% of the population reported regular intake of vitamin D supplements, with females comprising 71.1% of this subgroup. Furthermore, an additional 20.4% was using multivitamin products.

The distribution of the baseline characteristics across the four different datasets used for the four outcomes was generally similar (Supplemental Table S2), with the exception of a lower proportion of CHD (2.4%) observed in the dataset used for the CIMT.

### 3.2. Association of Vitamin D Exposure with Hypercalcemia

The cross-sectional associations of 25(OH)D level status and vitamin D supplement use with hypercalcemia are shown in Table 2. Compared to vitamin D sufficiency (25(OH)D between 50–<100 nmol/L), high vitamin D serum status (25(OH)D ≥ 100 nmol/L) was not associated with an increased prevalence of hypercalcemia. However, both vitamin supplement use and multivitamin use were associated with significantly increased odds of hypercalcemia at 46% and 11%, respectively.

To investigate, whether the hypercalcemia cases could be caused by a vitamin D overdose, we compared the distribution of 25(OH)D concentrations among users of vitamin D supplements with and without the presence of hypercalcemia (Supplemental Figure S1). The median 25(OH)D level of individuals with hypercalcemia (59.6, IQR: 44.9; 72.1, nmol/L) was not statistically significantly different (*p* = 0.14, Wilcoxon rank-sum test) from the median 25(OH)D level of individuals without hypercalcemia (57.4, IQR: 43.3; 71.0, nmol/L). Thus, the observed association between vitamin D supplement use and increased hypercalcemia risk is independent of the 25(OH)D levels.

Subgroup analyses for the association of high 25(OH)D status of ≥100 nmol/L and vitamin D supplement use with hypercalcemia did not show relevant differences between age groups, sexes, and kidney function (p_interaction_ > 0.05, Supplemental Tables S3–S5).

### 3.3. Association of Vitamin D Exposure with Kidney Stones

In the longitudinal analyses assessing the association of high vitamin D status, vitamin D supplement use, or multivitamin use with kidney stones, no statistically significant associations were observed (Table 3). In contrast, vitamin D deficiency and insufficiency were associated with a 7% and 11% increased risk of kidney stones in the fully adjusted model 2, respectively, but only the finding for vitamin D insufficiency was statistically significant.

Subgroup analyses showed that vitamin D supplement use and multivitamin use were associated with 23% and 11% decreased risk of kidney stones among younger participants, respectively. Other findings regarding the associations of high 25(OH)D levels ≥ 100 nmol/L and vitamin D supplement use with kidney stones across age groups, sexes, and kidney functions were comparable to the results of the main analysis (p_interaction_ > 0.05, Supplemental Tables S6–S8).

### 3.4. Association of Vitamin D Exposure with PASI and CIMT

No statistically significant associations were observed between high 25(OH)D levels, vitamin D use, or multivitamin use and either abnormal PASI or CIMT (Table 4). In contrast, vitamin D insufficiency was associated with a 6% increased risk of abnormal PASI levels in the full model. The association for vitamin D deficiency was the same (6% risk increase) but not statistically significant.

### 3.5. Dose–Response Association of 25(OH)D Levels with the Study Outcomes

**Figure 2:**
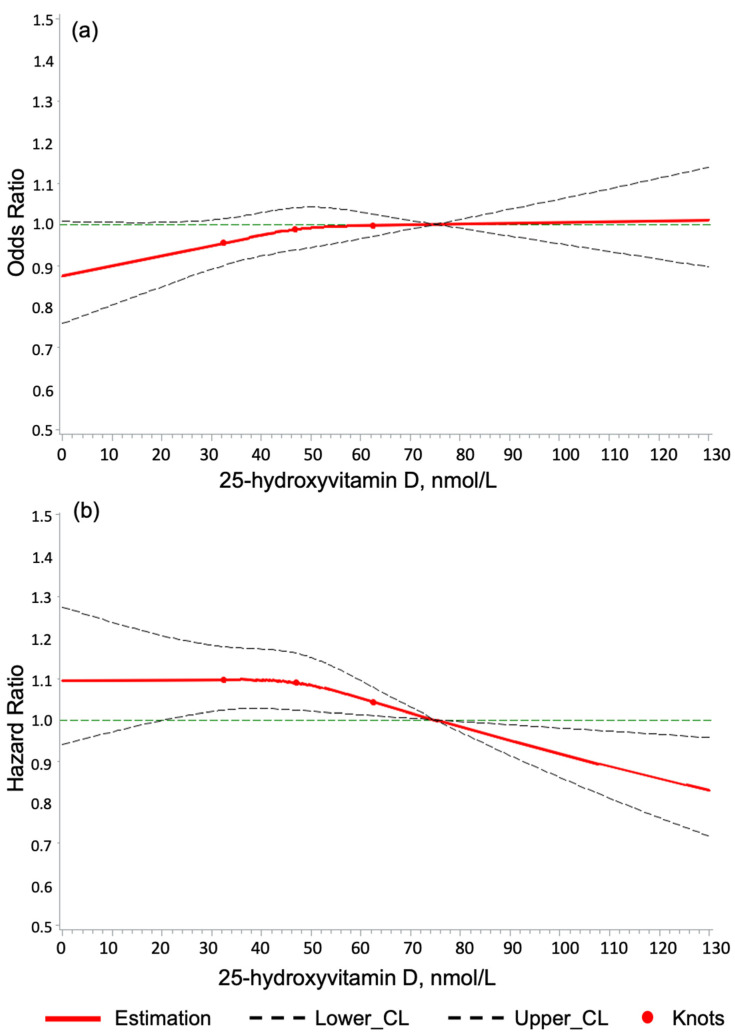
The dose–response relationship of serum 25-hydroxyvitamin D concentration with (**a**) hypercalcemia (cross-sectional analysis) and (**b**) kidney stones (longitudinal analysis). Notes: 3 knots were used and located at the 25th, 50th, and 75th serum 25-hydroxyvitamin D percentile, and the 75 nmol/L was used as the reference. Horizontal lines represent the odds ratio or hazard ratio of 1. Solid lines are estimates of the odds ratio or hazard ratios, and dashed lines are their 95% confidence intervals. Knots are represented by dots. The models are adjusted for all covariates included (n = 49, see legend of Table 2).

**Figure 3:**
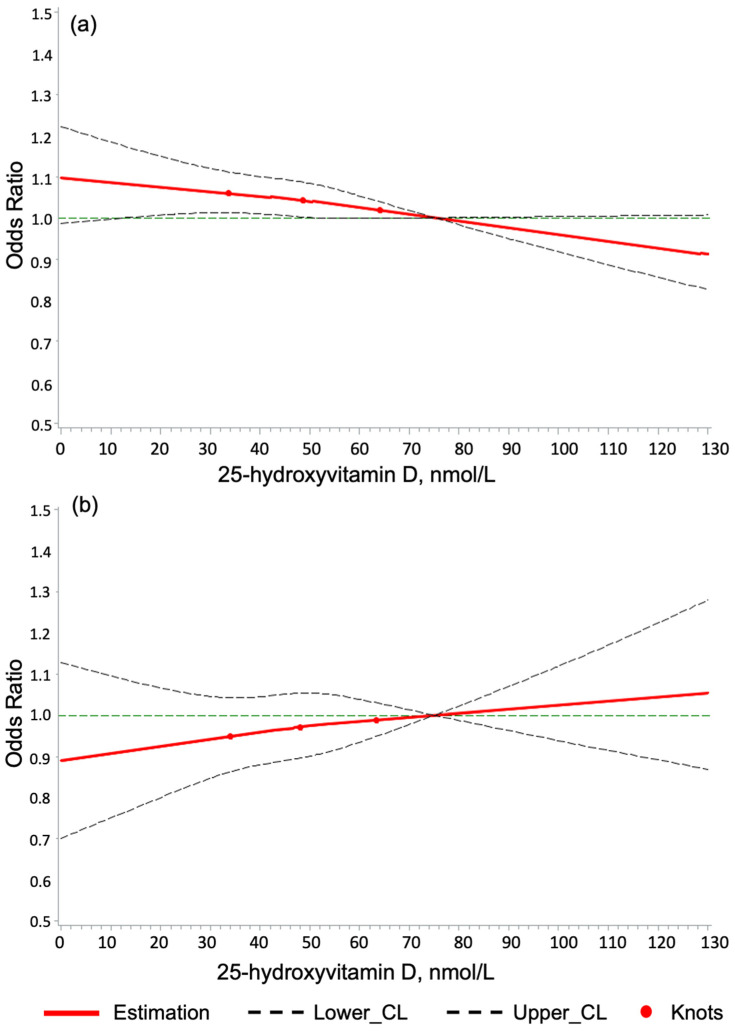
The dose–response relationship of serum 25-hydroxyvitamin D concentration with (**a**) pulse wave arterial stiffness index and (**b**) carotid intima medial thickness. Notes: 3 knots were used and located at the 25th, 50th, and 75th serum 25-hydroxyvitamin D percentile, and the 75 nmol/L was used as the reference. Horizontal lines represent the odds ratio of 1. Solid lines are estimates of odds ratios, and dashed lines are their 95% confidence intervals. Knots are represented by dots. The models are adjusted for all covariates included (n = 49, see legend of Table 2).

The RCS curves depicting the dose–response associations of serum 25(OH)D concentration and hypercalcemia, kidney stones, PASI, and CIMT did not reveal that 25(OH)D levels exceeding 100 nmol/L were associated with an increased risk of any adverse events of the study interest compared to the reference value of 75 nmol/L (Figure 2 and Figure 3).

In contrast, although statistical significance was not reached, there was a slightly decreased hypercalcemia risk for 25(OH)D levels below 50 nmol/L (Figure 2a). Furthermore, 25(OH)D levels > 80 nmol/L) were significantly associated with a reduced risk of kidney stones (Figure 2b). Similar to the dose–response curve for kidney stones, 25(OH)D levels > 80 nmol/L might be associated with lower odds for and adversely high PASI, but this association did not reach statistical significance (Figure 3a). Lastly, no dose–response association of 25(OH)D levels with the CIMT was detected because confidence intervals were wide (Figure 3b).

## 4. Discussion

### 4.1. Summary of the Study Findings

This study leveraged the data from 445,493 middle-aged and older adults from the UK Biobank to investigate the associations of high 25(OH)D levels and vitamin D supplement use with potential adverse outcomes, including hypercalcemia, kidney stones, and adversely high PASI and CIMT-two surrogate parameters of atherosclerosis. A high 25(OH)D level of ≥100 nmol/L did not show an association with any of the study outcomes in models comprehensively adjusted for 49 covariates. In contrast, we observed cross-sectional associations of low 25(OH)D levels in the vitamin D deficiency/insufficiency range with adversely high PASI and a longitudinal association with an increased risk of kidney stones. Both regular vitamin D use and multivitamin supplement use were associated with a higher prevalence of hypercalcemia but neither with atherosclerosis nor future kidney stones. In addition, the observed association was improbable due to vitamin D overdosing, as no statistically significant difference was observed between the distributions of 25(OH)D concentration in vitamin D users with and without hypercalcemia.

### 4.2. Vitamin D and Hypercalcemia

As of present, no meta-analysis is available for the association between high 25(OH)D concentrations and hypercalcemia. The finding of our study is consistent with observations from a single study that investigated the prevalence of hypercalcemia among 282,932 patients from a Turkish hospital, encompassing 495 individuals with notably elevated 25(OH)D levels exceeding 88 ng/mL (equivalent to 220 nmol/L) [38]. Similar to our results, the study from Turkey did not show a statistically significant association between high 25(OH)D levels and blood calcium levels [38].

However, a Mendelian randomisation study using genome-wide data from the UK Biobank showed an association between genetically predicted 25(OH)D levels and blood calcium levels [39]. This finding aligns with results from RCTs. A meta-analysis of 37 RCTs showed an increased risk of hypercalcemia in individuals receiving vitamin D supplements compared to those receiving a placebo (relative risk [RR], 95% CI: 1.54, 1.09; 2.18) [40]. This effect estimate is consistent with the one from our study, which used extensive real-world data from the general population (OR, 95%CI: 1.46, 1.32; 1.62 for comparison of vitamin D supplement users and non-users). Another recent meta-analysis of 10 RCTs, including only trials with high-dose vitamin D supplementation (3200 to 4000 IU per day), showed an even higher risk of hypercalcemia (RR, 95% CI: 2.21, 1.26; 3.87) [41]. However, the association between vitamin D supplement use and increased hypercalcemia risk observed in our study is unlikely to be due to vitamin D overdosing since none of the vitamin D users who were diagnosed with hypercalcemia exhibit excessively high 25(OH)D levels > 300 nmol/L, which was previously found to be safe concerning hypercalcemia events [42].

Given the comprehensive adjustment for numerous covariates, including the most common cause of hypercalcemia (i.e., hyperparathyroidism) in our analyses, the observed association might be explained by genetic factors, resulting in a higher sensitivity of the calcium metabolism to vitamin D and calcium co-supplementation. Studies suggest that the insufficient suppression of calcitriol in susceptible individuals may trigger excessive calcium absorption in the intestines when exposed to significant calcium intake [43,44]. Of note, in our study population, 60% of individuals who received vitamin D prescriptions from their general practitioners (GPs) received calcium prescriptions as well. It appears plausible to assume that the increased hypercalcemia risk observed in the UK Biobank cannot be explained by vitamin D supplement use alone but is rather attributable to the co-supplementation of vitamin D and calcium in individuals genetically susceptible to developing this adverse event.

### 4.3. Vitamin D and Kidney Stones

The association of vitamin D with kidney stones has frequently been explored. Mendelian randomisation studies showed an association of genetically predicted 25(OH)D with the risk of kidney stones [39,45]. In contrast, a meta-analysis of 32 observational studies involving 23,228 participants did not show a significant difference in serum 25(OH)D levels between individuals with and without nephrolithiasis, but their 1,25(OH)_2_D levels differed [46]. It is noteworthy that the majority of observational studies included in the meta-analysis were subject to limitations, such as a cross-sectional design, small sample sizes, and an inadequate adjustment of important vitamin D-related confounding factors [40,41,46,47,48]. Nevertheless, our longitudinal analysis was in agreement with the meta-analysis with regard to the absence of an association of high 25(OH)D levels with an increased risk of kidney stone formation. Likewise, there is no evidence from meta-analyses of RCTs that vitamin D supplementation increases the risk of kidney stones [40,41].

Contrary to the anticipated adverse impact of high 25(OH)D levels on kidney stones, we observed that low 25(OH)D levels were statistically significantly, yet weakly, associated with an increased risk of kidney stones. This finding could be supported by prior studies reporting a notable prevalence of vitamin D deficiency in stone formers. For instance, a case-control study involving 884 patients with idiopathic calcium nephrolithiasis compared to 967 controls revealed a higher prevalence of 25(OH)D levels < 50 nmol/L (56%) in stone formers than in the controls (44%; *p* < 0.001) [49]. Similarly, another case-control study examining 239 calcium stone formers vs. 127 controls yielded a comparable pattern (28% vs. 15.7%; *p* = 0.009) [50].

### 4.4. Vitamin D and PASI

In our study, we did not find evidence indicating an elevated risk of an adversely increased PASI associated with high 25(OH)D concentrations. Conversely, we observed a significant association between low 25(OH)D levels and an adversely increased PASI. Consistent with our findings, an observational study conducted among 544 healthy participants aged 20–79 years in the US showed an association between vitamin D insufficiency and increased arterial stiffness [51]. Similar findings regarding the association between 25(OH)D levels and arterial stiffness were also observed in two out of three smaller-scale observational studies conducted in Canadian, Turkish, and Chinese populations (n = 123–175) [52,53,54,55].

A positive effect of adequate 25(OH)D levels on arterial stiffness is also supported by evidence from RCTs. A meta-analysis of 9 RCTs showed an improvement in arterial stiffness following vitamin D supplementation, particularly in subgroup analyses targeting individuals with vitamin D deficiency, with study durations of ≥ 4 months and daily doses of vitamin D_3_ ≥ 2000 IU [56].

### 4.5. Vitamin D and CIMT

A meta-analysis incorporating data from 17 observational studies revealed an inverse association between 25(OH)D concentrations and the continuous CIMT scale [57]. However, upon pooling the risk estimates from another three observational studies using ORs for adverse CIMT scales, no significant association was observed between serum vitamin D status and the risk of CIMT (OR, 95% CI: 0.96, 0.74; 3.86) [57]. This observation is further corroborated by several other observational studies that likewise failed to identify such an association [58,59], which is in agreement with our findings from the large UK Biobank. Although there is some evidence for the efficacy of vitamin D supplementation on a reduction in CIMT from RCTs, this needs to be interpreted with caution because it is only based on 3 RCTs involving a total of 99 participants [57].

### 4.6. Dose–Response Relationships of 25(OH)D Concentration with the Study Outcomes

To our knowledge, this study marks the first exploration of dose–response relationships between 25(OH)D concentrations and calcium metabolism-related safety outcomes. No U-shaped associations were observed, which was consistent with the reported effect estimates for vitamin D deficiency, vitamin D insufficiency, and high vitamin D status.

### 4.7. Strengths and Limitations

To our knowledge, this is the largest study investigating the potential adverse events of regular vitamin D supplementation in the general population. The utilisation of data from the large UK Biobank provided the investigation sufficient statistical power to detect potential associations despite the low frequency of these events in the general population. Moreover, the UK Biobank offers valuable information on the usage of vitamin D supplements obtained over the counter, a practice more common than prescription-based supplementation. Thus, allowing for the investigation of actual vitamin D supplement usage patterns. Furthermore, the comprehensive adjustment for up to 50 covariates, including vitamin D-specific factors, controlled for confounding to a large extent, although residual confounding can never be completely excluded in observational studies.

However, this study also has limitations. The UK Biobank is subject to the well-known “healthy volunteer” bias, potentially influencing the estimation of disease prevalence (e.g., prevalence of diabetes mellitus) [60]. Nevertheless, the relative risk estimates derived from exposure-outcome associations are expected to be less susceptible to this bias. Additionally, the study lacks information regarding the dosage, chemical properties, and frequency of intake (e.g., daily, weekly, or monthly) of the vitamin D supplements used. Unfortunately, urinary calcium levels were not assessed in the UK Biobank, which precluded adding hypercalciuria as an endpoint to this investigation.

Regarding the potential adverse events studied, we did not look into other potential consequences of hypercalcemia in addition to kidney stones or atherosclerosis (e.g., cardiac arrhythmias, hyporeflexia, and muscle weakness). These are areas for future research. Furthermore, we employed ICD-10 code N20 to identify kidney stones, which includes but is not limited to calcium-based stones. However, since approximately 80% of kidney stones are calcium-based [61], we think that the inclusion of other stone types in the endpoint definition will have influenced the results to a minimal extent.

Lastly, the findings of this study could be primarily generalisable to individuals of European ancestry residing in regions without widespread vitamin D food fortification. Variations in results might be observed in regions with food fortification, differing levels of sun exposure, and non-White ethnicities.

## 5. Conclusions

In the large, population-based UK Biobank cohort, high 25(OH)D levels did not demonstrate any associations with potential adverse calcium metabolism-related outcomes, including hypercalcemia, kidney stones, and surrogate parameters of atherosclerosis. Although regular vitamin D supplement use was associated with a higher prevalence of hypercalcemia, it was neither associated with atherosclerosis nor future kidney stones. Moreover, we provide additional analyses showing that the hypercalcemia cases are likely not caused by vitamin D supplementation alone. We assume that these cases have been caused by vitamin D and calcium co-supplementation in specifically genetically susceptible individuals.

We conclude that the use of multivitamin and vitamin D supplements in the real-life setting in the UK is practiced safely. This finding is not surprising when keeping in mind that 10,000 IU (=250 µg) vitamin D_3_/day is the lowest dose in clinical trials at which adverse effects were observed and that 4000 IU (=100 µg) vitamin D_3_/day is the official tolerable upper intake level for adults in the European Union [62]. Such high intakes are likely rarely present in the general population.

## Figures and Tables

**Table 1:** Baseline characteristics of the UK Biobank study population with vitamin D-related data (N = 445,493).

Variables	N (%) ^a^/Median (IQR)
**Sex**, n (%)	
Female	238,942 (53.6)
Male	206,551 (46.4)
**Age (years)**	
Mean (SD)	56.5 (8.1)
Median (IQR)	58 (50; 63)
**BMI**, n (%)	
<18.5	2285 (0.5)
18.5–<25	145,608 (32.8)
25–<30	188,111 (42.4)
≥30	107,814 (24.3)
**Smoking**, n (%)	
Never	244,481 (54.9)
Ever	200,868 (45.1)
**Alcohol consumption** ^b^, n (%)	
Abstainer	137,944 (31.0)
Low	178,258 (40.0)
Medium	75,677 (17.0)
High	53,614 (12.0)
**Hypertension**, n (%)	119,924 (26.9)
**Diabetes**, n (%)	22,264 (5.0)
**CHD**, n (%)	20,841 (4.7)
**eGFR (ml/min/1.73 m^2^)**, n (%)	
≥90	264,715 (59.5)
60–<90	170,102 (38.2)
<60	10,135 (2.3)
**Hyperparathyroidism**, n (%)	583 (0.1)
**Calcium (mmol/L)**, Median (IQR)	2.37 (2.31; 2.43)
**Hypercalcemia, n (%)**	6325 (1.6)
**Kidney stones (during follow-up)**, n (%)	5097 (1.1)
**PASI (m/s)**, Median (IQR)	9.0 (6.9; 11.1)
**Adversely high PASI** ^c^, n (%)	11,473 (7.6)
**Average of mean CIMT (mm)**, Median (IQR)	0.67 (0.60; 0.76)
**Adversely high CIMT > 0.9 mm**, n (%)	2713 (6.2)
**No. of chronic diseases**, Median (IQR)	2 (1; 3)
**25(OH)D concentration (nmol/L)**, Median (IQR)	46.9 (32.4; 62.5)
**Vitamin D status, n (%)**	
Vitamin D deficiency (<30 nmol/L)	93,406 (21.0)
Vitamin D insufficiency (30–<50 nmol/L)	152,925 (34.3)
Vitamin D sufficiency (50–<100 nmol/L)	192,488 (43.2)
High vitamin D status (≥100 nmol/L)	6674 (1.5)
**Regular vitamin supplement use**, n (%)	
None	335,562 (75.3)
Multivitamin ± minerals product	90,752 (20.4)
Vitamin D-specific medication	19,179 (4.3)

Note: 25(OH)D: 25-hydroxyvitamin D, BMI: body mass index, CHD: coronary heart disease, CIMT: carotid intima-medial thickness, eGFR: estimated glomerular filtration rate, IQR: interquartile range, PASI: pulse wave arterial stiffness index, SD: standard deviation. ^a^ Denominators in proportion calculation do not include missing values. ^b^ Alcohol consumption: Low: Women >0–19.99 g of ethanol per day (g/d) or men > 0–39.99 g/d; Medium: Women 20–39.99 g/d or men 40–59.99 g/d; High: Women ≥ 40 g/d or men ≥ 60 g/d. ^c^ Based on calculated age-specific cut-off values.

**Table 2:** Associations of vitamin D serum status with hypercalcemia prevalence (cross-sectional analysis).

Vitamin D Exposure	Hypercalcemia Prevalence			
	N_total_	N_case_ (%)	Odds Ratio (95%CI)	
			Model 1 ^a^	Model 2 ^b,c^
**Vitamin D serum status, (25(OH)D, nmol/L)**				
Deficiency (<30)	85,776	1214 (1.4)	**0.93 (0.87; 0.99)**	0.94 (0.87; 1.02)
Insufficiency (30–<50)	140,013	2144 (1.5)	0.96 (0.91; 1.02)	0.98 (0.93; 1.04)
Sufficiency (50–<100)	175,302	2871 (1.6)	Ref	Ref
High status (≥100)	6094	96 (1.6)	1.01 (0.82; 1.24)	0.98 (0.80; 1.21)
**Vitamin supplement users**				
Non-users	306,849	4470 (1.5)	Ref	Ref
Multivitamin user	82,793	1374 (1.7)	**1.08 (1.01; 1.15)**	**1.11 (1.04; 1.18)**
Vitamin D user	17,543	481 (2.7)	**1.57 (1.43; 1.73)**	**1.46 (1.32; 1.62)**

Abbreviation: 25(OH)D: 25-hydroxyvitamin D, CI: confidence interval, Ref: reference. Numbers in bold represent results of statistical significance. ^a^ Model 1 is adjusted for age and sex. ^b^ For vitamin D serum status, model 2 is adjusted for age, sex, skin colour, latitude of study centre and calendar month of attending the assessment centre, socio-economic factors (education, Townsend deprivation index, no of individuals in household, and household income), life-style factors (smoking, alcohol consumption, physical activity, frequency of visiting friends/family and consumption of oily fish, cereal, processed meat, milk, bread and spread), and vitamin D-specific factors (time spend outdoors in summer and winter, ease of skin tanning, use of sun screen/UV protection, and solarium/sunlamp use), weight variables (body mass index and waist circumference), diseases and disease symptoms (hyperparathyroidism, diabetes, stroke, coronary heart disease, chronic obstructive pulmonary disease, osteoporosis, arthritis, gout, Parkinson, depressed mood, and tiredness/lethargy), biomarkers (estimated glomerular filtration rate, HbA1c, HDL cholesterol, systolic blood pressure, diastolic blood pressure, C-reactive protein, forced expiratory volume in 1-s, and hand grip strength), and general health status (no. of drugs, no. of chronic diseases, disability, and general self-rated health). ^c^ For vitamin supplement use, model 2 is adjusted for age, sex, skin colour, latitude of study centre and calendar month of attending the assessment centre, socio-economic factors (Townsend deprivation index, no of individuals in household, and household income), life-style factors (smoking, alcohol consumption, physical activity, venturesome personality, frequency of visiting friends/family) and vitamin D-specific factors (consumption of oily fish, processed meat, milk, bread, spread, time spend outdoors in summer, ease of skin tanning, use of sun screen/UV protection, and solarium/sunlamp use), weight variables (body mass index and waist circumference), diseases and disease symptoms (hyperparathyroidism, cancer, hypertension, stroke, coronary heart disease, chronic obstructive pulmonary disease, asthma, osteoporosis, fractured in last 5 years, arthritis, gout, diabetes, hypothyroidism, chronic fatigue syndrome, tiredness/lethargy in last 2 weeks, dementia, Parkinson, and depressed mood), biomarkers (estimated glomerular filtration rate, C-reactive protein), general health status (disability, general self-rated health, and no. of drugs), and medication intake (low dose aspirin, lipid-lowering drugs, and anti-depression drugs).

**Table 3:** Associations of vitamin supplement use with kidney stone incidence (longitudinal analysis).

Vitamin D Exposure	Kidney Stone Incidence			
	N_total_	N_case_ (%)	Hazard Ratio (95%CI)	
			Model 1 ^a^	Model 2 ^b^
**Vitamin D serum status, (25(OH)D, nmol/L)**				
Deficiency (<30)	92,063	1172 (1.3)	**1.28 (1.19; 1.38)**	1.07 (0.98; 1.16)
Insufficiency (30–<50)	150,657	1859 (1.2)	**1.20 (1.13; 1.28)**	**1.11 (1.04; 1.19)**
Sufficiency (50–<100)	189,875	1994 (1.1)	Ref	Ref
High status (≥100)	6594	72 (1.1)	1.03 (0.81; 1.30)	1.03 (0.81; 1.30)
**Vitamin supplement users**				
Non-users	330,625	3980 (1.2)	Ref	Ref
Multivitamin user	89,638	925 (1.0)	**0.92 (0.85; 0.99)**	0.96 (0.89; 1.03)
Vitamin D user	18,926	192 (1.0)	0.97 (0.84; 1.12)	0.95 (0.82; 1.10)

Abbreviation: 25(OH)D: 25-hydroxyvitamin D, CI: confidence interval, Ref: reference. Numbers in bold represent results of statistical significance. ^a^ Model 1 adjusted for age and sex. ^b^ For covariates of model 2, please see legend of Table 2.

**Table 4:** Cross-sectional associations of vitamin D serum status as well as vitamin supplement use with pulse wave arterial stiffness index and carotid intima-medial thickness (cross-sectional analysis).

Vitamin D Exposure	PASI				CIMT			
	N_total_	N_case_ (%)	Odds Ratio (95%CI)		N_total_	N_case_ (%)	Odds Ratio (95%CI)	
			Model 1 ^a^	Model 2 ^b^			Model 1 ^a^	Model 2 ^b^
**Vitamin D serum status, (25(OH)D, nmol/L)**								
Deficiency (<30)	28,842	2519 (8.7)	**1.22** **(1.16; 1.28)**	1.06 (0.99; 1.12)	8091	456 (5.6)	1.09 (0.97; 1.22)	0.97 (0.85; 1.11)
Insufficiency (30–<50)	49,880	3960 (7.9)	**1.13** **(1.08; 1.18)**	**1.06** **(1.01; 1.11)**	15,327	918 (6.0)	1.02 (0.93; 1.12)	0.96 (0.87; 1.06)
Sufficiency (50–<100)	68,827	4813 (7.0)	Ref	Ref	19,844	1297 (6.5)	Ref	Ref
High status (≥100)	2568	181 (7.1)	0.98 (0.84; 1.15)	1.04 (0.89; 1.21)	696	42 (6.0)	0.90 (0.65; 1.24)	0.86 (0.62; 1.20)
**Vitamin supplement use**								
Non-users	112,249	8764 (7.8)	Ref	Ref	32,925	2045 (6.2)	Ref	Ref
Multivitamin user	30,863	2203 (7.1)	0.94 (0.89; 0.98)	0.98 (0.94; 1.03)	9246	555 (6.0)	1.05 (0.95; 1.16)	1.06 (0.96; 1.17)
Vitamin D user	7005	506 (7.2)	1.02 (0.93; 1.13)	1.08 (0.98; 1.19)	1787	113 (6.3)	1.02 (0.84; 1.25)	1.13 (0.92; 1.38)

Abbreviation: 25(OH)D: 25-hydroxyvitamin D, CI: confidence interval, CIMT: carotid intima medial thickness, PASI: pulse wave arterial stiffness index, Ref: reference. Numbers in bold represent results of statistical significance. ^a^ Model 1 adjusted for age and sex. ^b^ Model 2 adjusted for all covariates (see legend of Table 2).

## Data Availability

The study used data from the UK Biobank. Publicly available data are accessible to researchers via an open application on https://www.ukbiobank.ac.uk/register-apply/ (accessed on 10 June 2024).

## Supplementary Materials

The following supporting information can be downloaded at https://www.mdpi.com/article/10.3390/nu16142251/s1, Figure S1: Distribution of serum 25-hydroxyvitamin D concentration among regular vitamin D supplement users stratified by hypercalcemia status; Table S1: Complete list of baseline characteristics of the population analyzed (N = 445,493); Table S2: Overviews of baseline characteristics for study population in the four analytical datasets; Table S3: Cross-sectional associations of vitamin D serum status and vitamin supplement use with hypercalcemia, subgroup analysis by age groups; Table S4: Cross-sectional associations of vitamin D serum status and vitamin supplement use with hypercalcemia, subgroup analysis by sex; Table S5: Cross-sectional associations of vitamin D serum status as well as vitamin supplement use with hypercalcemia, subgroup analyses by kidney function; Table S6: Longitudinal associations of vitamin D serum status and vitamin supplement use with kidney stones, subgroup analysis by age groups; Table S7: Longitudinal associations of vitamin D serum status as well as vitamin supplement use with kidney stones, subgroup analysis by sex; Table S8: Longitudinal associations of vitamin D serum status as well as vitamin supplement use with kidney stones, subgroup analyses by kidney function.
