# Supplementary material for: The Safety Profile of Vitamin D Supplements Using Real-World Data from 445,493 Participants of the UK Biobank: Slightly Higher Hypercalcemia Prevalence but Neither Increased Risks of Kidney Stones nor Atherosclerosis
Source: Nutrients. 2024 Jul 12;16(14):2251. doi: 10.3390/nu16142251 (PMC11279740; doi:10.3390/nu16142251)
Supplement: Supplementary file 1 [file nutrients-16-02251-s001.zip › nutrients-3079998-supplementary.pdf]

Supplemental Materials

for

Safety Profile of Vitamin D Supplements Using Real-World Data from 445,493 Participants of the UK Biobank: Slightly Higher Hypercalcemia Prevalence but Neither Increased Risks of Kidney Stones nor Atherosclerosis

Table of Contents

Table S1. Complete list of baseline characteristics of the population analyzed (N=445,493) 2

Table S2. Overviews of baseline characteristics for study population in the four analytical datasets 7

Table S3. Cross-sectional associations of vitamin D serum status and vitamin supplement use with hypercalcemia, subgroup analysis by age groups 9

Table S4. Cross-sectional associations of vitamin D serum status and vitamin supplement use with hypercalcemia, subgroup analysis by sex 10

Table S5. Cross-sectional associations of vitamin D serum status as well as vitamin supplement use with hypercalcemia, subgroup analyses by kidney function 11

Table S6. Longitudinal associations of vitamin D serum status and vitamin supplement use with kidney stones, subgroup analysis by age groups 12

Table S7. Longitudinal associations of vitamin D serum status as well as vitamin supplement use with kidney stones, subgroup analysis by sex 13

Table S8. Longitudinal associations of vitamin D serum status as well as vitamin supplement use with kidney stones, subgroup analyses by kidney function 14

Figure S1. Distribution of serum 25-hydroxyvitamin D concentration among regular vitamin D supplement users stratified by hypercalcemia status 15

Table S1. Complete list of baseline characteristics of the population analyzed (N=445,493)

Variables	N (%) [a]/ Median (IQR)
**SOCIO-DEMOGRAPHIC/ -ECONOMIC FACTORS**	
**Age (years), median (IQR)**	58 (50; 63)
**Sex, n (%)**	
Female	238,942 (53.6)
Male	206,551 (46.4)
**Education (years), median (IQR)**	11 (10; 17)
**Townsend deprivation index (points), median (IQR)**	-0.3 (-0.7; 0.4)
**No. of individuals in household, n (%)**	
1	81,563 (18.3)
2	206,572 (46.4)
3-4	130,470 (29.3)
≥ 5	23,885 (5.3)
**Annual household income (£), n (%)**	
< 18,000	85,742 (19.2)
18,000 - < 30,999	96,749 (21.7)
31,000- < 51,999	100,058 (22.5)
52,000 - < 100,000	78,640 (17.7)
≥ 100,000	21,000 (4.7)
**LIFE-STYLE FACTORS**	
**Smoking, n (%)**	
Never	244,481 (54.9)
Occasionally	63,247 (14.2)
Regularly	137,621 (30.9)
**Alcohol consumption (g ethanol/d), n (%)**	
Abstainer	137,944 (31.0)
Women 0 - < 20 / men 0 - < 40	178,258 (40.0)
Women 20 - < 40 / men 40 - < 60	75,677 (17.0)
Women ≥ 40 / men ≥ 60	53,614 (12.0)
**Venturesome personality, n (%)**	
No	313,093 (70.3)
Yes	115,706 (26.0)
**Total physical activity (hours/day), n (%)**	
≤ 1	63,786 (14.3)
≤ 2	140,699 (31.6)
> 2	142,047 (31.9)
**Frequency of visiting friends/family, n (%)**	
Almost daily	59,760 (13.4)
2-4 times/week	158,268 (35.5)
Once/week	136,111 (30.6)
Once every few months/rare	51,555 (11.6)
**Oily fish consumption, n (%)**	
Never/ less than once a week	195,508 (43.9)
At least once a week	247,484 (55.6)
**Cereal consumption (bowls/week), n (%)**	
Never	75,841 (17.0)
< 7	198,082 (44.5)
≥ 7	170,066 (38.2)

Variables	N (%) [a]/ Median (IQR)
**Processed meat intake, n (%)**	
Never/ less than once a week	176,398 (39.6)
At least once a week	268,147 (60.2)
**Milk consumption, n (%)**	
Never/rarely	14,590 (3.3)
Occasionally/regularly	430,583 (96.7)
**Spread consumption, n (%)**	
Never/rarely	48,201 (10.8)
Butter	160,664 (36.1)
Margarine/others	235,855 (52.9)
**Preferred bread type, n (%)**	
White	113,435 (25.5)
Wholemeal/wholegrain/brown	316,220 (70.9)
DISEASES & DISEASE SYMPTOMS	
**Diabetes, n (%)**	
No	423,131 (94.9)
Yes	22,264 (5.1)
**Stroke, n (%)**	
No	439,410 (98.6)
Yes	5,984 (1.3)
**CHD, n (%)**	
No	424,553 (95.3)
Yes	20,841 (4.7)
**COPD, n (%)**	
No	443,907 (99.6)
Yes	1,487 (0.3)
**Hypertension, n (%)**	
No	325,480 (73.1)
Untreated hypertension	33,253 (7.5)
Treated hypertension	86,671 (19.5)
**Asthma, n (%)**	
No	393,732 (88.4)
Yes	51,662 (11.6)
**Osteoporosis, n (%)**	
No	434,431 (97.5)
Yes	10,963 (2.5)
**Fracture in last 5 years, n (%)**	
No	401,340 (90.1)
Yes	41,918 (9.4)
**Arthritis, n (%)**	
No	398,918 (89.5)
Yes	46,476 (10.4)
**Gout, n (%)**	
No	438,211 (98.4)
Yes	7,183 (1.6)
**Parkinson, n (%)**	
No	444,460 (99.8)
Yes	934 (0.2)
**Depressed mood in last 2 weeks, n (%)**	
≤ half the days	404,447 (90.8)

Variables	N (%) [a]/ Median (IQR)
> half the days	21,023 (4.7)
**Tiredness/lethargy in last 2 weeks, n (%)**	
≤ half the days	377,274 (84.7)
> half the days	54,403 (12.2)
**Chronic fatigue syndrome, n (%)**	
No	443,479 (99.5)
Yes	1,915 (0.4)
**Hypothyroidism, n (%)**	
No	423,940 (95.2)
Yes	21,454 (4.8)
**Dementia, n (%)**	
No	442,754 (99.4)
Yes	2,640 (0.6)
**Cancer, n (%)**	
No	410,621 (92.2)
Yes	33,570 (7.5)
**Hyperparathyroidism, n (%)**	
No	444,910 (99.9)
Yes	583 (0.1)
**BIOMARKERS**	
**BMI (kg/m^2), n (%)**	
Underweight, < 18.5	2,285 (0.5)
Low normal weight, 18.5 - <20	8,188 (1.8)
High normal weight, 20 - < 25	137,420 (30.8)
Overweight: 25 - < 30	188,111 (42.2)
Obesity class I: 30 - < 35	77,278 (17.3)
Obesity class II: 35 - < 40	22,021 (4.9)
Obesity class III: ≥ 40	8,515 (1.9)
**Waist circumference (cm), median (IQR)**	90 (80; 99)
**eGFR (ml/min/1.73 m^2), n (%)**	
≥ 90	264,715 (59.4)
< 90	180,237 (40.5)
**HbA$_{1c}$, (%), n (%)**	
< 6	411,465 (92.4)
6 - < 6.5	17,488 (3.9)
6.5 - < 7	6,204 (1.4)
7 - < 8	5,972 (1.3)
≥ 8	4,364 (1.0)
**HDL cholesterol (mg/dl), n (%)**	
< 40	89,800 (20.1)
≥ 40	355,693 (79.8)
**SBP (mmHg), n (%)**	
< 140	236,879 (53.2)
140 - < 160	140,521 (31.5)
160 - < 180	53,988 (12.1)
≥ 180	14,105 (3.2)
**DBP (mmHg), n (%)**	
< 90	339,609 (76.2)
90 - < 100	80,476 (18.1)
≥ 100	25,408 (5.7)

Variables	N (%) [a]/ Median (IQR)
**C-reactive protein (mg/L), n (%)**	
< 1	177,546 (39.9)
≥ 1	267,947 (60.1)
**FEV1 (L), median (IQR)**	2.8 (2.3; 3.3)
**Hand grip strength (Kg), median (IQR)**	31 (24; 40)
**GENERAL HEALTH**	
**Disability (%)**	
No	416,119 (93.4)
Yes	25,799 (5.8)
**General self-reported health, n (%)**	
Excellent	73,626 (16.5)
Good	257,527 (57.8)
Fair	92,895 (20.9)
Poor	19,507 (4.4)
**No of chronic diseases, median (IQR)**	2 (1; 3)
**No of drugs, median (IQR)**	2 (0; 4)
**Low-dose aspirin use, n (%)**	
No	382,319 (85.8)
Yes	63,075 (14.2)
**Lipid-lowering drugs use, n (%)**	
No	365,816 (82.1)
Yes	79,585 (17.9)
**Anti-depressants use, n (%)**	
No	416,037 (93.4)
Yes	29,357 (6.6)
**VITAMIN D SPECIFIC FACTORS**	
**Latitude of study center (per 1°), median (IQR)**	53.0 (51.5; 53.8)
**Month of attending the study center (month of blood draw)**	
1	30,516 (6.8)
2	35,121 (7.9)
3	43,433 (9.7)
4	38,830 (8.7)
5	46,372 (10.4)
6	46,028 (10.3)
7	38,137 (8.6)
8	33,952 (7.6)
9	32,617 (7.3)
10	38,241 (8.6)
11	37,335 (8.4)
12	24,911 (5.6)
**Time spent outdoors in summer (h/day), n (%)**	
<1	18,616 (4.2)
1-2	130,269 (29.2)
3-4	138,749 (31.1)
5-6	84,309 (18.9)
≥ 7	48,338 (10.9)
**Time spent outdoors in winter (h/day), n (%)**	

Variables	N (%) [a]/ Median (IQR)
<1	83,763 (18.8)
1-2	238,767 (53.6)
3-4	63,525 (14.3)
≥ 5	34,082 (7,6)
**Skin color, n (%)**	
Very fair	34,021 (7.6)
Fair	299,581 (67.2)
Olive	81,675 (18.3)
Brown	8,248 (1.9)
Black	12,428 (2.8)
Unknown	3,364 (0.8)
**Ease of skin tanning, n (%)**	
Very tanned	94,016 (21.1)
Moderately tanned	173,061 (38.8)
Mildly/occasionally tanned	91,909 (20.6)
Never tan, only burn	74,631 (16.8)
**Sun screen/UV protection use, n (%)**	
Never/rarely	44,849 (10.1)
Sometimes	148,262 (33.3)
Most of times	157,360 (35.3)
Always	91,671 (20.6)
Do not go out in sunshine	2,666 (0.6)
**Solarium/sunlamp use (times per year), n (%)**	
Never	399,596 (89.7)
< 1	21,586 (4.8)
1 - 6	10,720 (2.4)
7 - 12	4,624 (1.0)
> 12	4,390 (1.0)

Abbreviations: BMI: body mass index CHD: coronary heart disease, COPD: chronic obstructive pulmonary disease, DBP: diastolic blood pressure, eGRF: estimated Glomerular filtration rate, FEV1: Forced expiratory volume in 1-second, IQR: interquartile range, SBP: systolic blood pressure

[a] Denominators in proportion calculations contain missing values.

Table S2. Overviews of baseline characteristics for study population in the four analytical datasets

Variables	Dataset for analysis of			
	Hypercalcemia N=407,185	Kidney stone N= 439,189	PASI N=150,117	CIMT N=43,958
	N (%) [a] /Median (IQR)			
**Sex,** n (%)				
Female	216,671 (53.2)	237,079 (54.0)	80,118 (53.4)	22,710 (51.7)
Male	190,514 (46.8)	202,110 (46.0)	69,999 (46.6)	21,248 (48.3)
**Age (years)**				
Mean (SD)	56.5 (8.1)	56.4 (8.1)	56.7 (8.2)	55.1 (7.6)
Median (IQR)	58 (50; 63)	58 (50; 63)	58 (50; 63)	56 (49; 61)
**BMI,** n (%)				
< 18.5	2,092 (0.5)	2,268 (0.5)	768 (0.5)	195 (0.4)
18.5 - <25	132,408 (32.5)	144,148 (32.9)	49,001 (32.7)	17,241 (39.3)
25 - < 30	172,089 (42.3)	185,336 (42.2)	63,333 (42.2)	18,834 (42.8)
≥30	99,049 (24.3)	105,802 (24.1)	36,838 (24.5)	7631 (17.3)
**Smoking,** n (%)				
Never	223,218 (54.8)	241,248 (54.9)	83,003 (55.3)	26,626 (60.6)
Ever	183,833 (45.0)	197,799 (45.1)	67,050 (44.7)	17,327 (39.4)
**Alcohol consumption** [b], n (%)				
Abstainer	126,017 (30.9)	135,739 (30.9)	49,074 (32.7)	10,442 (23.8)
Low	163,194 (40.1)	175,571 (40.0)	59,889 (39.9)	19,244 (43.8)
Medium	69,070 (17.0)	74,854 (17.0)	23,981 (16.0)	8,559 (19.5)
High	48,904 (12.0)	53,025 (12.1)	17,173 (11.4)	5,713 (13.0)
**Hypertension,** n (%)	109,824 (27.0)	117,488 (26.7)	40,838 (27.2)	8,554 (19.4)
**Diabetes,** n (%)	20,356 (5.1)	21,662 (4.9)	8,209 (5.5)	1,092 (2.5)
**CHD,** n (%)	19,089 (4.7)	20,311 (4.6)	6,774 (4.5)	1,051 (2.4)
**eGFR (ml/min/1.73 m²),** n (%)				
≥ 90	242,500 (59.6)	261,252 (59,5)	87,250 (58.1)	27,130 (61.7)
60-< 90	155,290 (38.1)	167,534 (38.1)	59,184 (39.4)	16,232 (36.9)
< 60	9,157 (2.2)	9,868 (2.2)	3,487 (2.3)	539 (1.2)
**Hypercalcemia**	6,325 (1.6)	-	-	-
**Kidney stones during follow-up,** n (%)	-	5,097 (1.2)	-	-
**PASI,** Median (IQR)	-	-	9.0 (6.9; 11.1)	-
**Adversely high PASI** [c], n (%)	-	-	11,473 (7.6)	-
**Average of mean CIMT,** Median (IQR)	-	-	-	0.67 (0.60; 0.76)
**Adversely high CIMT > 0.9 mm,** n (%)	-	-	-	2,713 (6.2)
**No. of chronic diseases,** Median (IQR)	2 (1;3)	2 (1;3)	2 (1;3)	1 (0;2)
**25(OH)D concentration (nmol/L),** Median (IQR)	46.8 (32.3; 62.4)	46.9 (32.4; 62.5)	48.5 (33.7; 64.0)	48.1 (33.9; 63.4)
**Vitamin D status,** n (%)				
Deficiency (<30 nmol/L)	85,776 (21.1)	92,063 (21)	28,842 (19.2)	8091 (18.4)
Insufficiency (30- <50 nmol/L)	140,013 (34.4)	150,657 (34.3)	49,880 (33.2)	15,327 (34.9)
Sufficiency (50 - < 100 nmol/L)	175,302 (43.1)	189,875 (43.2)	68,827 (45.8)	19,844 (45.1)
High status (≥ 100 nmol/L)	6,094 (1.5)	6,594 (1.5)	2,568 (1.7)	696 (1.6)
**Regular vitamin supplement use,** n (%)				
No	306,849 (75.4)	330,625 (75.3)	112,249 (74.8)	32,925 (74.9)
Multivitamins +/-minerals	82,793 (20.3)	89,638 (20.4)	30,863 (20.6)	9,246 (21.0)
Vitamin D	17,543 (4.3)	18,926 (4.3)	7,005 (4.7)	1,787 (4.1)

Abbreviations: 25(OH)D: 25-hydroxyvitamin D, BMI: body mass index, CHD: coronary heart disease, CIMT: carotid intima-medial thickness, eGFR: estimated glomerular filtration rate, IQR: interquartile range, PASI: pulse wave arterial stiffness index, SD: standard deviation.

[a] Denominators in proportion calculations contain missing values.

[b] Alcohol consumption: Low: Women > 0-19.99 grams of ethanol per day (g/d) or men > 0-39.99 g/d; Medium: Women 20-39.99 g/d or men 40-59.99 g/d; High: Women ≥ 40g/d or men ≥ 60 g/d.

[c] Based on calculated age specific cut-off values.

Table S3. Cross-sectional associations of vitamin D serum status and vitamin supplement use with hypercalcemia, subgroup analysis by age groups

Vitamin D exposure	Age, 40 - 59 years [a] N=231,560			Age, 60 - 69 years [b] N=175,625			p-interaction [c]
	N_{total}	N_{case} (%)	OR (95%CI)	N_{total}	N_{case} (%)	OR (95%CI)	
**Vitamin D serum status, (25[OH]D, nmol/L)**							
**Deficiency (<30)**	55,930	676(1.2)	0.94(0.84,1.05)	29,846	538(1.8)	0.95(0.85,1.06)	0.93
**Insufficiency (30-<50)**	81,079	1,060(1.3)	1.00(0.92,1.10)	58,934	1,084(1.8)	0.96(0.89,1.05)	0.97
**Sufficiency (50-<100)**	91,177	1,237(1.4)	Ref	84,125	1,634(1.9)	Ref	-
**High status (≥100)**	3,374	37(1.1)	0.80(0.58,1.12)	2,720	59(2.2)	1.13(0.87,1.48)	0.36
**Vitamin supplement use**							
**Non-users**	175,023	2,141(1.2)	Ref	131,826	2,329(1.8)	Ref	-
**Multivitamin user**	48,331	679(1.4)	**1.12(1.02,1.22)**	34,462	695(2.0)	**1.10(1.01,1.20)**	0.21
**Vitamin D user**	8,206	190(2.3)	**1.55(1.33,1.82)**	9,337	291(3.1)	**1.39(1.22,1.58)**	0.26

Abbreviations: 25(OH)D: 25-hydroxyvitamin D, CI: confidence interval, HR: hazard ratio, Ref: reference.
Bold print: statistically significant (p<0.05).
All covariates were adjusted in the model (see the legend of Table 2)
[a] including as small minority of study participants aged 37-39 years (n=6)
[b] including as small minority of study participants aged 70-73 years (n=1,947)
[c] Age was considered as continuous variable when calculating interaction terms.

Table S4. Cross-sectional associations of vitamin D serum status and vitamin supplement use with hypercalcemia, subgroup analysis by sex

Vitamin D exposure	Female			Male			p-interaction
	N_{total}	N_{case} (%)	OR (95%CI)	N_{total}	N_{case} (%)	OR (95%CI)	
**Vitamin D serum status, (25[OH]D, nmol/L)**							
**Deficiency (<30)**	45,362	794 (1.8)	**0.89 (0.81, 0.98)**	40,414	420 (1.0)	1.02 (0.88, 1.17)	**<.001**
**Insufficiency (30-<50)**	74,311	1,492 (2.0)	0.96 (0.90, 1.03)	65,702	652 (1.0)	1.00 (0.90, 1.12)	0.19
**Sufficiency (50-<100)**	93,904	2,065 (2.2)	Ref	81,398	806 (1.0)	Ref	-
**High status (≥100)**	3,094	67 (2.2)	1.02 (0.79, 1.31)	3,000	29 (1.0)	0.93 (0.64, 1.36)	0.97
**Vitamin supplement use**							
**Non-users**	154,733	2,983 (1.9)	Ref	152,116	1,487 (1.0)	Ref	-
**Multivitamin user**	49,521	1,017 (2.1)	**1.10 (1.02, 1.18)**	33,272	357 (1.1)	**1.14 (1.01, 1.28)**	0.63
**Vitamin D user**	12,417	418 (3.4)	**1.50 (1.34, 1.67)**	5,126	63 (1.2)	1.20 (0.93, 1.56)	0.59

Abbreviation: 25(OH)D: 25-hydroxyvitamin D, CI: confidence interval, HR: hazard ratio, Ref: reference.
Bold print: statistically significant ($p<0.05$).
All covariates were adjusted in the model (see the legend of Table 2)

Table S5. Cross-sectional associations of vitamin D serum status as well as vitamin supplement use with hypercalcemia, subgroup analyses by kidney function

Vitamin D exposure	eGFR < 60 ml/min/1.73m²		eGFR 60-<90 ml/min/1.73m²		eGFR ≥ 90 ml/min/1.73m²		p-interaction
	N_{case} (%)	OR (95%CI) [a]	N_{case} (%)	OR (95%CI) [a]	N_{case} (%)	OR (95%CI) [a]	
**Vitamin D serum status, (25[OH]D, nmol/L)**							
**Deficiency**	42 (2.2)	**0.64 (0.43; 0.96)**	469 (1.7)	0.97 (0.86; 1.10)	701 (1.2)	0.95 (0.86; 1.06)	0.72
**Insufficiency**	103 (3.3)	1.03 (0.78; 1.37)	892 (1.7)	0.96 (0.88; 1.05)	1,146 (1.4)	1.00 (0.92; 1.09)	0.27
**Sufficiency**	130 (3.3)	Ref	1,338 (1.8)	Ref	1,394 (1.4)	Ref	-
**High status**	5 (2.3)	0.67 (0.26; 1.70)	43 (1.7)	0.93 (0.68; 1.27)	47 (1.4)	1.05 (0.78; 1.42)	0.75
**Vitamin supplement use**							
**Non-users**	216 (3.0)	Ref	1,961 (1.7)	Ref	2,284 (1.3)	Ref	-
**Multivitamin user**	44 (3.1)	1.00 (0.71; 1.41)	588 (2.0)	**1.15 (1.04; 1.26)**	736 (1.4)	1.08 (0.99; 1.18)	0.21
**Vitamin D user**	20 (4.3)	1.09 (0.66; 1.79)	193 (2.9)	**1.35 (1.15; 1.58)**	268 (2.6)	**1.59 (1.39; 1.82)**	0.15

Abbreviation: 25(OH)D: 25-hydroxyvitamin D, CI: confidence interval, eGRF: estimated Glomerular filtration rate, OR: odds ratio, Ref: reference.
Bold print: statistically significant ($p<0.05$).
[a] Model adjusted for all covariates (see legend of Table 2)

Table S6. Longitudinal associations of vitamin D serum status and vitamin supplement use with kidney stones, subgroup analysis by age groups

Vitamin D exposure	Age, 40 - 59 years [a] N=250,696			Age, 60 - 69 years [b] N=188,493			p-interaction[c]
	N_{total}	N_{case} (%)	HR (95%CI)	N_{total}	N_{case} (%)	HR (95%CI)	
**Vitamin D serum status, (25[OH]D, nmol/L)**							
**Deficiency (<30)**	60,077	713 (1.2)	1.07 (0.95, 1.20)	31,986	459 (1.4)	1.07 (0.94, 1.21)	0.75
**Insufficiency (30-<50)**	87,610	1,024 (1.2)	**1.13 (1.03, 1.24)**	63,047	835 (1.3)	1.09 (0.99, 1.20)	0.35
**Sufficiency (50-<100)**	99,329	954 (1.0)	Ref	90,546	1,040 (1.2)	Ref	-
**High status (≥100)**	3,680	35 (1.0)	1.00 (0.71, 1.40)	2,914	37 (1.3)	1.06 (0.76, 1.47)	0.78
**Vitamin supplement use**							
**Non-users**	189,248	2,167 (1.2)	Ref	141,377	1,813 (1.3)	Ref	-
**Multivitamin user**	52,564	486 (0.9)	**0.89 (0.81, 0.99)**	37,074	439 (1.3)	1.04 (0.93,1.16)	0.06
**Vitamin D user**	8,884	73 (0.8)	**0.77 (0.61, 0.98)**	10,042	119 (1.2)	1.10 (0.91,1.34)	0.12

Abbreviation: 25(OH)D: 25-hydroxyvitamin D, CI: confidence interval, HR: hazard ratio, Ref: reference.
Bold print: statistically significant ($p<0.05$).
All covariates were adjusted in the model (see the legend of Table 2)
[a] including as small minority of study participants aged 37-39 years (n=5)
[b] including as small minority of study participants aged 70-73 years (n=2,088)
[c] Age was considered as continuous variable when calculating interaction terms.

Table S7. Longitudinal associations of vitamin D serum status as well as vitamin supplement use with kidney stones, subgroup analysis by sex

Vitamin D exposure	Female			Male			p-interaction
	N_{total}	N_{case} (%)	HR (95%CI)	N_{total}	N_{case} (%)	HR (95%CI)	
**Vitamin D serum status, (25[OH]D, nmol/L)**							
**Deficiency (<30)**	49,295	455 (0.9)	**1.18 (1.02, 1.35)**	42,768	717 (1.7)	1.02 (0.91, 1.13)	0.02
**Insufficiency (30-<50)**	81,292	657 (0.8)	**1.15 (1.03, 1.29)**	69,365	1,202 (1.7)	**1.09 (1.01, 1.19)**	0.54
**Sufficiency (50-<100)**	103,117	685 (0.7)	Ref	86,758	1,309 (1.5)	Ref	-
**High status (≥100)**	3,375	28 (0.8)	1.23 (0.84, 1.80)	3219	44 (1.4)	0.93 (0.69, 1.26)	0.19
**Vitamin supplement use**							
**Non-users**	169,252	1,333 (0.8)	Ref	161,373	2,647 (1.6)	Ref	-
**Multivitamin user**	54,301	374 (0.7)	0.91 (0.81, 1.02)	35,337	551 (1.6)	0.99 (0.90, 1.09)	0.21
**Vitamin D user**	13,526	118 (0.9)	1.05 (0.87, 1.28)	5400	74 (1.4)	0.82 (0.65, 1.03)	0.08

Abbreviation: 25(OH)D: 25-hydroxyvitamin D, CI: confidence interval, HR: hazard ratio, Ref: reference.
Bold print: statistically significant ($p<0.05$).
All covariates were adjusted in the model (see the legend of Table 2)

Table S8. Longitudinal associations of vitamin D serum status as well as vitamin supplement use with kidney stones, subgroup analyses by kidney function

Vitamin D exposure	eGFR < 60 ml/min/1.73m²		eGFR 60-<90 ml/min/1.73m²		eGFR ≥ 90 ml/min/1.73m²		p-interaction
	N_{case} (%)	HR (95%CI) [a]	N_{case} (%)	HR (95%CI) [a]	N_{case} (%)	HR (95%CI) [a]	
**Vitamin D serum status, (25[OH]D, nmol/L)**							
**Deficiency**	49 (2.4)	1.57 (0.99, 2.50)	382 (1.3)	1.01 (0.88, 1.17)	740 (1.2)	1.09 (0.98, 1.22)	0.18
**Insufficiency**	57 (1.7)	1.27 (0.86, 1.89)	664 (1.2)	1.01 (0.91, 1.12)	1,136 (1.3)	**1.18 (1.08, 1.29)**	0.21
**Sufficiency**	52 (1.2)	Ref	866 (1.1)	Ref	1,071 (1.0)	Ref	-
**High status**	4 (1.7)	1.42 (0.50, 4.06)	36 (1.3)	1.11 (0.79, 1.56)	32 (0.9)	0.92 (0.64, 1.31)	0.23
**Vitamin supplement use**							
**Non-users**	132 (1.7)	Ref	1,535 (1.2)	Ref	2,307 (1.2)	Ref	-
**Multivitamin user**	20 (1.3)	0.86 (0.53, 1.40)	351 (1.1)	1.03 (0.91, 1.15)	553 (1.0)	0.92 (0.84, 1.01)	0.37
**Vitamin D user**	10 (2.0)	1.42 (0.73, 2.76)	62 (0.9)	0.85 (0.66, 1.10)	119 (1.1)	0.99 (0.82, 1.19)	0.60

Abbreviation: 25(OH)D: 25-hydroxyvitamin D, CI: confidence interval, eGRF: estimated Glomerular filtration rate, OR: odds ratio, Ref: reference.
Bold print: statistically significant ($p<0.05$).
[a] Model adjusted for all covariates (see legend of Table 2)

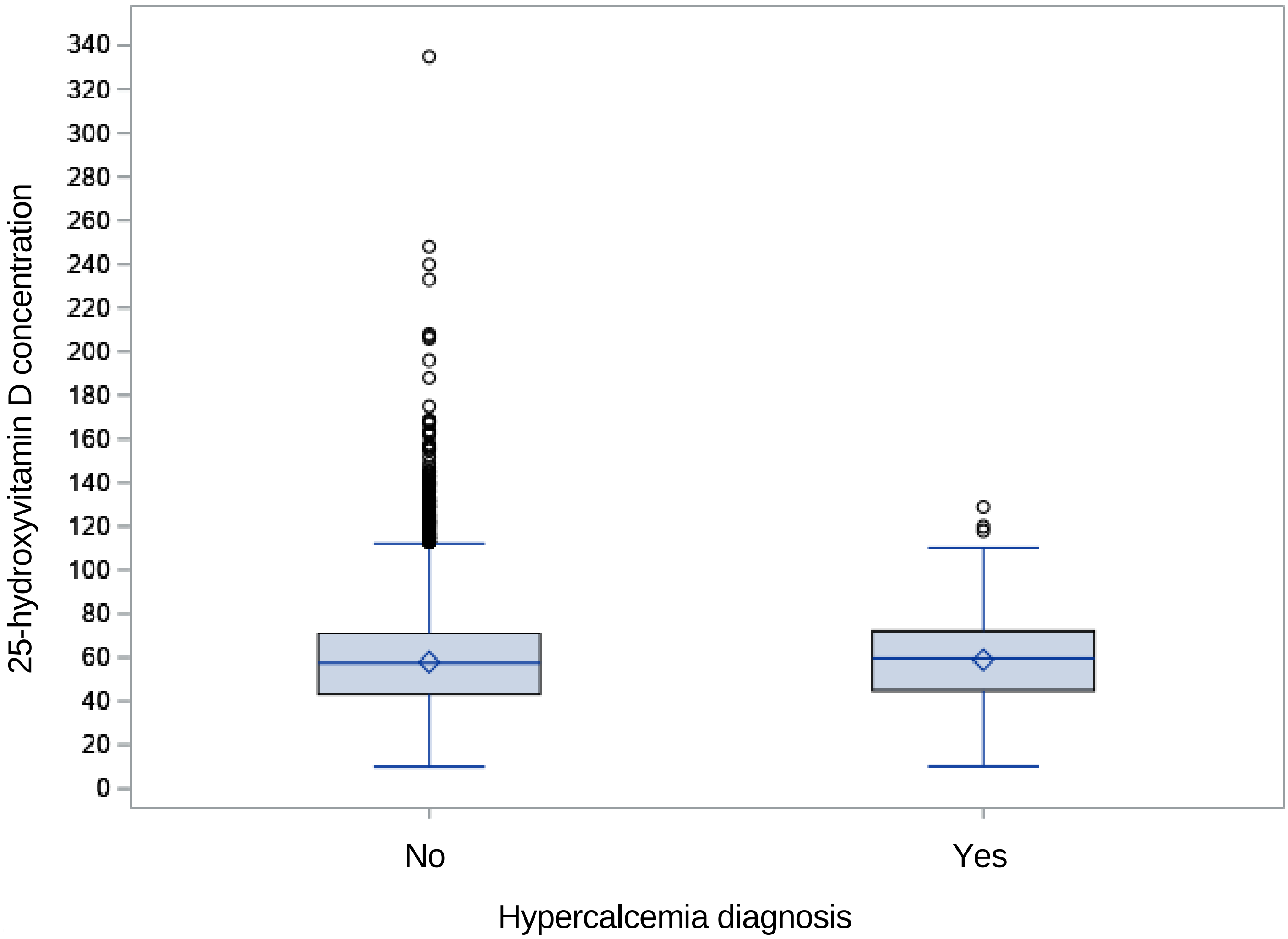

Figure S1. Distribution of serum 25-hydroxyvitamin D concentration among regular vitamin D supplement users stratified by hypercalcemia status

Notes: The sample sizes are as follows: $n_{(total)}$ =17,543, $n_{(without\ hypercalcemia)}$=17,062, $n_{(with\ hypercalcemia)}$=481. The Wilcoxon rank sum test for comparing the median 25-hydroxyvitamin D levels in the groups result was p=0.14.
